# Assessment of the adherence to and costs of the prophylaxis protocol for venous thromboembolism

**DOI:** 10.6061/clinics/2019/e1143

**Published:** 2019-08-13

**Authors:** Marcela Forgerini, Fabiana Rossi Varallo, Alice Rosa Alves de Oliveira, Tales Rubens de Nadai, Patrícia de Carvalho Mastroianni

**Affiliations:** I Departamento de Farmacos e Medicamentos, Faculdade de Ciencias Farmaceuticas, Universidade Estadual de Sao Paulo (UNESP), Araraquara, SP, BR; II Departamento de Ciencias Farmaceuticas, Faculdade de Ciencias Farmaceuticas de Ribeirao Preto, Universidade de Sao Paulo, Ribeirao Preto, SP, BR; III Departamento de Cirurgia e Anatomia, Faculdade de Medicina de Ribeirao Preto, Universidade de Sao Paulo, Ribeirao Preto, SP, BR

**Keywords:** Venous Thromboembolism, Pharmaceutical Economics, Quality Indicators, Patient Safety, Safety Management

## Abstract

**OBJECTIVES:**

Evaluate adherence to the therapeutic prophylaxis protocol for venous thromboembolism (VTE) as well as the costs of this practice.

**METHODS:**

A descriptive and cross-sectional study was conducted at a State General Hospital in Brazil through reports of drug dispensions, prescriptions and risk stratification of patients. Adherence to the VTE prophylaxis protocol was monitored. The tests for VTE diagnosis measured the adherence to therapeutic prophylaxis treatment, and the purchase prices of the drugs went into the calculation of drug therapy costs. The level of adherence to prescriptions for VTE prophylaxis in the hospital was classified as “adherence”, “non-adherence” and “justified non-adherence” when compared with the protocol.

**RESULTS:**

Protocol adherence was observed for 50 (30.9%) patients, and non-adherence was observed for 63 (38.9%) patients, generating an additional cost of $180.40/month. Justified non-adherence in 49 (30.2%) patients generated $514.71/month in savings due to a reduction in the number of daily administrations of unfractionated heparin while still providing an effective method for preventing VTE. Twenty-six patients stratified as having medium to high risk of VTE who did not receive prophylaxis were identified, generating $154.41 in savings. However, these data should be evaluated with caution since the risks and outcomes associated with not preventing VTE outweigh the economy achieved from not prescribing a drug when a patient needs it. The only case of VTE identified during the study period was related to justified non-adherence to the protocol.

**CONCLUSION:**

The protocol is based on scientific evidence that describes an effective therapy to prevent VTE. However, the protocol should be updated because the justifications for non-adherence are based on scientific evidence, and this justified non-adherence generates savings and yields effective disease prevention.

## INTRODUCTION

The term venous thromboembolism (VTE) is used to denote the combination of two pathologies: deep venous thrombosis (DVT) and pulmonary thromboembolism (PTE), which is the more serious clinical condition 1. During the hospitalization period, half of patients are at risk of developing VTE, and the incidence is higher among surgical patients 2,3.

Despite the high incidence (approximately 104 to 183/100,000/year) and severity of VTE, it is a preventable cause of morbidity and mortality in hospitalized patients 4 and is highly preventable when adequate prophylactic measures are performed 5. Therefore, the application of evidence-based prophylactic measures in surgical patients in the hospital setting may reduce the number of individuals affected by VTE and its consequences 6. However, even with well-established guidelines, VTE prophylaxis is not always properly practiced 3,7.

Unfractionated heparin (UFH) and low-molecular weight heparin (LMWH) are among the drugs used in the prophylaxis protocol, and both correspond to approximately 6% of the monthly cost of purchasing drugs in the hospital setting 8.

In this context, quality management tools that monitor adherence to established protocols are relevant in promoting the rational use of prophylactic therapy, aimed at reducing the incidence of DVT and PTE at the lowest cost and with methods based on scientific evidence.

Thus, we aimed to evaluate adherence to the criteria of the care protocol for prophylactic VTE therapy and the costs generated by this practice in a single hospital.

## METHODS

### Study design and setting

This is a descriptive and cross-sectional study carried out at Américo Brasiliense State Hospital (HEAB), a medium-sized general hospital linked to the Public Health System (SUS) of Brazil.

The project was approved by the Scientific Project Analysis Committee of the hospital.

### Patients

The VTE care protocol determines measures of and therapeutic prophylaxis for moderate risk and high risk of VTE. For moderate risk, subcutaneous UFH 5,000 IU / 0.25 mL twice daily or a subcutaneous LMWH-filled syringe 20 mg / 0.2 mL once per day is recommended; for high risk, a subcutaneous UFH ampoule 5,000 IU / 0.25 mL three times a day or LMWH 40 mg / 0.4 mL once daily is recommended 8. Mechanical prophylaxis is indicated when inpatients are unable to use UFH or LMWH due to renal impairment, overcoagulation, or old age, among other conditions.

The prophylactic drug therapy for VTE should be administered only to patients stratified as moderate- and high-risk and according to some criteria established in the protocol (Table 1).

**Figure 1:**
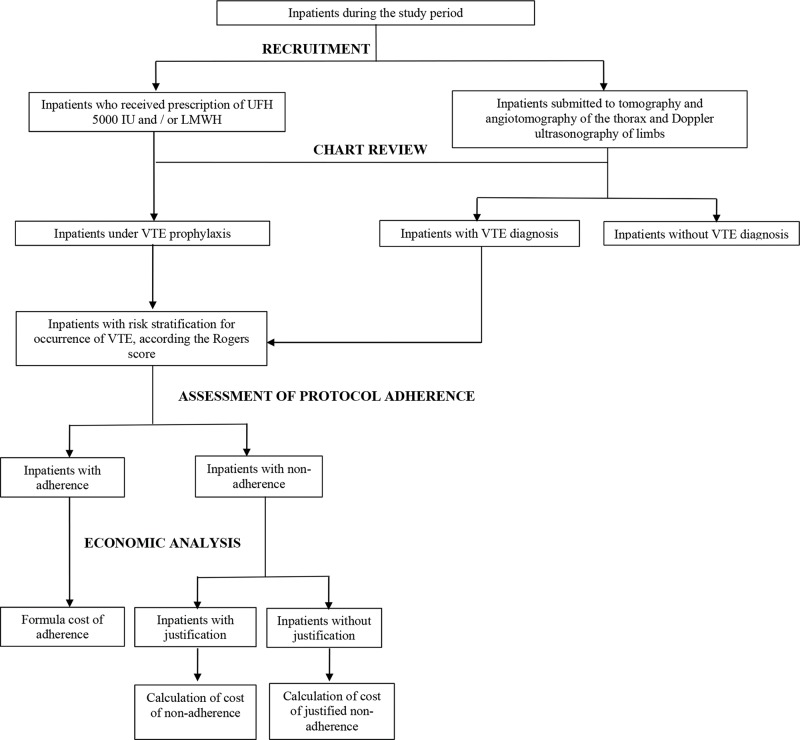
Recruitment, chart review, evaluation of protocol adherence and economic analysis of patients with venous thromboembolism.

Participants were recruited if they experienced two triggers: 1) hospital prescription of UFH 5000 IU and/or LMWH 20 mg or 40 mg; and 2) inpatient status as well as submission to imaging exams (tomography, angiotomography of the thorax and Doppler ultrasonography of limbs). Therefore, inpatients met the inclusion criteria when they received prescriptions of UFH 5000 IU and/or LMWH 20 mg or 40 mg for VTE prophylaxis or the diagnosis of VTE (Figure 1).

The medical records of patients prescribed UFH 5000 IU or a full-dose of LMWH (1 mg/kg) associated with oral anticoagulants were excluded from this study. This association is used for the treatment of PTE and has resulted in a reduction in hospital stay 10. However, for inpatients with a diagnosis of VTE confirmed by imaging exams, we performed a review of records to assess whether these patients received prophylaxis according to our institutional protocol.

Chart review was carried out for enrolled inpatients to assess the adherence to prescription of VTE prophylaxis according to the risk stratification described in the institutional protocol and the costs associated with adherence and non-adherence to the drug therapy protocol.

Risk stratification was performed at HEAB by an algorithm based on the Rogers score 9. The algorithm assigns a score to each risk factor, and the sum of the assigned points stratifies the patient into low, medium- or high-risk categories. The risk factors investigated included advanced age, DVT or PTE for more than two years, obesity, prolonged surgery with 60 minutes under anesthesia, and paralysis of a lower limb, among others 9.

The Rogers score is often used as a standard because of its simplicity of application and reproducibility, since the laboratory tests, such as sodium and albumin levels, required for the Rogers score are usually collected as part of routine patient hospitalization. This score is also used by the Clinical Hospital of the Medical School of the University of São Paulo (USP), and although the American College of Chest Physicians (ACCP) supports the use of the Caprini score, this score requires more complex exams that may make it difficult to execute and deploy.

### Data and measures

Data were collected three months after protocol implementation. Data collection was performed through the hospital pharmacy dispensing report and was complemented with the information on the dossier of the received prophylactic therapy (UFH 5000 IU and/or LMWH 20 mg or 40 mg) in addition to the clinical conditions of the patient, including the Rogers score risk stratification analysis.

To perform chart review and identify and collect the data of patients stratified into the medium- and high-risk categories and who did not receive prophylactic therapy, the Athos - Hospital Assistance System of HEAB was consulted. Athos is a consolidated electronic medical records system with the information and anamnesis of a multiprofessional team in addition to the results of clinical and laboratory tests.

The results of tomography and angiotomography of the thorax and Doppler ultrasonography of limbs were consulted during the patients' hospitalization to collect the cases of VTE and to evaluate whether the patient received the drug therapy described in the protocol.

The descriptive variables studied were the level of adherence to the protocol criteria ("adherence", "*non-*adherence" or "justified *non-*adherence"), the prescribers, the ward where patients were hospitalized and received or should have received drug therapy, and the cost of therapy according to the type of adherence to the protocol, and an economic evaluation was performed.

*"*Adherence*"* was considered the fulfillment of all the criteria established in the VTE care protocol; *"non-*adherence*"* was non-adherence to at least one criterion established in the protocol without any justification; and *"justified non-*adherence*"* was non-adherence to the protocol but with justification based on scientific evidence. A patient hospitalized for several days who had more than one prescription was stratified into the category *"*adherence*"* when all the prescriptions complied with the protocol. Failure to comply with the protocol on any patient prescription prompted placement in the *"non-*adherence*"* group. Finally, when the patient received at least one prescription that did not comply with the protocol, with justification, and none without justification, this was considered *"justified non-*adherence *".*

The dichotomous variable studied was the presence or absence of DVT/PTE, according to patient adherence to the protocol criteria.

### Economic analysis

The currency used for the costs in the present study was the dollar. For the economic evaluation of each type of adherence, a *"referential* adherence*"* was established, that is, the theoretical cost if the protocol had been followed in full.

The cost of *"referential* adherence*"* was calculated using the following formula:

*C_ra_* = *V_estimated_* x *I_estimated_* x *N_nc_*

C_ra_: cost of referential adherence.

V_estimated_: unit value of the drug to be used according to the protocol (UFH 5,000 IU or LMWH 20 mg or 40 mg).

I_estimated_: dosing interval that should be used according to protocol (two or three times daily).

N_nc_: absolute frequency of hospitalization.

The same formula and reasoning were adopted for the calculation of the cost of non-adherence (C_na_) and the cost of justified non-adherence (C_naj_).

To evaluate the difference between the cost of adherence and the cost of non-adherence to the protocol (with or without justification), the following formula was used:

*E* = *C_ra_*–*C_na_*

In the case of justified non-adherence, the formula was:

*E* = *C_ra_*–*C_naj_*

E: economic evaluation.

C_ra_: cost of referential adherence.

C_na:_ cost of non-adherence.

C_naj_: cost of justified non-adherence.

### Statistical analysis

The collected data were tabulated in a spreadsheet using Microsoft Excel^®^ 2013. Data were analyzed through descriptive statistics to present the simple and relative frequencies of the variables.

## RESULTS

A total of 162 patients eligible for the study were identified. We analyzed 1208 prescriptions, of which 123 included therapeutic prophylaxis for VTE. In addition, 39 prescriptions were associated with patients stratified as having medium or high risk and who did not receive prophylaxis.

The most frequent diagnoses of patients who received VTE prophylaxis in the study period were bacterial and viral pneumonia, chronic obstructive pulmonary disease, stroke, congestive heart failure and malignant neoplasms.

Adherence to the VTE protocol was identified in 50 patients (30.9%) (Table 2). Non-adherence to one or more protocol criteria was identified in 63 patients (38.9%), of whom 26 were stratified as having medium or high risk and did not receive prophylaxis without any contraindications. Justified non-adherence was observed for 49 patients (30.2%) [UFH twice daily for high-risk patients] (Table 3).

Regarding the prescriptions analyzed [1208], adherence to the protocol was observed in 457 prescriptions (37.8%) (Table 2). In 231 (19.1%), there was non-adherence to the protocol without justification. Non-adherence was justified for 520 prescriptions (43%) (Table 3).

The justified non-adherence generated a savings of $514.12/month; however, non-adherence without justification generated an additional cost of $180.20 in the analyzed month (Table 3).

No differences were observed in the frequency of non-adherence to the protocol for prophylactic VTE therapy according to the ward or prescriber. In addition, there was uniformity in practice among the 65 physicians responsible for the prescriptions analyzed.

From another perspective, there was only one record of VTE. A female patient, 63 years old, with moderate obesity, hypertension and dyslipidemia, was hospitalized at HEAB for neurological rehabilitation of hemorrhagic stroke. Throughout the entire hospitalization the patient received VTE prophylaxis, and in the first two days of hospitalization, the protocol was not fulfilled with justification. Subsequently, until hospital discharge, the protocol was fulfilled. However, on the 10th day, the patient evolved with respiratory arrest, was transferred to the intensive care unit with suspected acute myocardial infarction and was later diagnosed with PE.

Due to the recent hemorrhagic stroke, full anticoagulation was not performed. A vena cava filter was implanted in the patient, which effectively prevented mortality. After discharge (28 days of hospitalization), the patient was referred to a qualified hospital to begin full anticoagulation therapy.

## DISCUSSION

The incidence of PTE increased from 23/100.000/year in 1993 to 65/100.000 in 2012 12.

Studies with hospitalized patients have demonstrated that the incidence of VTE cases can vary between 0.52 and 0.97% 13,14. In the present study, only one case of VTE (PTE) was recorded (0.6%), similar to another study that identified only one case during the one-year experience reported after the implementation of the VTE prophylaxis protocol 15. The case also occurred with an obese female patient, 57 years old, after her hospital discharge 15. This low incidence may corroborate the hypothesis that VTE therapeutic prophylaxis is effective. Regarding the case of VTE, it was observed that the justified non-adherence to the prophylaxis protocol could not be considered the sole cause of the PTE. The patient, who received VTE prophylaxis for two days with reduction in the daily dose of UFH, already had some risk factors for the development of VTE. Hemorrhagic stroke, obesity and old age are important risk factors 16,17. In addition, hemorrhagic stroke may increase the risk of VTE by five times 18, and age over 40 years significantly increases the risk of VTE, which doubles every subsequent decade 16.

Furthermore, the causality between therapeutic ineffectiveness and justified non-adherence to the protocol was considered improbable despite a reasonable temporal relationship between the prophylaxis administration and the diagnosis of PTE, since the thrombotic event could be explained by other clinical factors 19.

As described in Table 2, the criterion "prescription of UFH 7,500 IU for morbidly obese patients" was not fulfilled for any patient. This was probably due to the absence of the 7,500 IU-compatible dosage in the Brazilian market, and dosage using two UFH 5,000 IU ampoules would generate waste.

The most recent Clinical Practice Guidelines of the ACCP recommend, in addition to HFN 7,500 IU three times daily, prophylactic treatment with LMWH 40 mg twice daily for these patients 20. Thus, it is recommended that the dosage for these patients be updated in the protocol so that they do not receive UFH subdoses and that there is no waste of the drug.

For moderately obese patients (body mass index <40), such as patients diagnosed with PTE, the ideal dosage of prophylactic therapy is not clearly defined due to the poor quality of evidence 20.

Another criterion of the protocol that was not followed, with justification, was the prescription of prophylaxis for patients with low risk. According to the ACCP guidelines, patients stratified as low risk should not receive therapeutic prophylaxis but should receive only mechanical prophylaxis 21. Therefore, it is considered important that the prescribing physician justify this practice in patient medical records when the application of prophylactic therapy to low-risk patients is deemed necessary.

Despite the lack of clear evidence in the literature regarding which drug is most cost-effective (UFH or LMWH), the choice between the two drugs may be related to local factors that affect acquisition costs 21. Because the acquisition of UFH is less expensive, the HEAB care protocol mostly recommends the use of UFH. However, other factors may interfere with this decision, such as the preference of the prescriber and the ease of administration in the patient 21. Therefore, it is suggested that the factors described in the protocol should be considered by the prescriber to avoid unnecessary hospital expenses.

Similarly, according to the ACCP guidelines, there is no robust evidence to show that administration of UFH three times a day confers a greater risk of bleeding or greater effectiveness in relation to administration of UFH twice daily 21. The low incidence of VTE demonstrated in this study strengthens this premise, since a significant proportion of the patients analyzed (30.2%) received prophylaxis with UFH twice daily. In addition, for the only case of VTE detected, the patient had other risk criteria that could explain the event.

The similarity between the numbers of patients who received the two types of prescriptions [three times a day (49) and twice a day (54)] suggests that prescribers were aware of recent evidence that shows no risk of bleeding with the use of UFH three times a day and no lowered effectiveness with the use of UFH twice daily. Furthermore, twice daily UFH administration, which generates a savings of $514.71/month, is more commonly acceptable and better tolerated by the patient than three daily injections 21. Therefore, the twice daily dosage is considered the best option because it corroborates with the rational use of drugs (use of drugs appropriate for clinical conditions, in doses appropriate to the individual needs, for an appropriate period and at the lowest cost) 22 and because there are no differences in safety or effectiveness. For this reason, we suggest updating the relevant healthcare protocol of HEAB based on scientific evidence, a task assigned to the Hospital's Pharmacy and Therapy Commission 23.

Although adherence to the protocol was only 30%, this was calculated before an educational intervention to promote behavioral changes and improve adherence to the protocol and before the update of the protocol to the highest evidentiary standard, according to the data obtained from protocol monitoring.

The monitoring of protocol adherence is an indicator of hospital healthcare assistance and consists of a quality tool and comprehensive management. Furthermore, despite the development of prevention and treatment strategies, VTE remains a burden on public health 24.

In this sense, the importance of the clinical pharmacy in the management of clinical protocols is emphasized because the professionals in this area have specialized therapeutic knowledge, being able to assist in the choice of adequate therapy and the duration of the therapy and to discuss the cost effectiveness of treatment options 25–27.

Thus, monitoring adherence to protocols has two benefits: one that contributes to patient safety, fostering the practice of prophylactic therapy that is based on scientific evidence and best suited to the clinical conditions of the patient; and one that aims at reducing costs for the institution since clinical protocols recommend the most cost-effective treatments 28.

The recruitment technique of this study was unable to identify patients at risk of VTE and who were not using prophylactic therapy because the medical records analysis was based on the recruitment of patients with LMWH or UFH prescriptions and with a diagnosis of VTE. We were unable to assess the use of mechanical devices because the hospital did not use electronic registration with the dispensing of these items. Thus, we were unable to identify patients who received a prescription for such devices. In addition, the short collection period may be an important limitation of this study because it did not allow the identification of seasonality, but it was possible to analyze all admissions in the study period.

Another limitation was that the economic analysis considered only the costs of drug therapy, disregarding other expenses arising from the patient's hospitalization and related complications.

## CONCLUSION

The prophylaxis protocol considered in this study is based on scientific evidence that describes drug therapies that are effective for VTE prevention. The highest cost of non-adherence to the protocol came from administration in low-risk-stratified patients who, according to the protocol, should not receive therapeutic prophylaxis. The prescription of prophylaxis in these cases is considered unnecessary and contributes to extra costs to the hospital.

The savings generated by non-adherence refers to non-administration of the drug to medium- and high-risk patients and/or the administration of underdoses to obese patients due to the lack of availability of the specialty dosage (7,500 IU). However, despite the savings generated, patients without prophylaxis have a higher risk of developing VTE. Thus, the costs associated with prophylaxis therapy should not outweigh the benefits associated with preventing negative outcomes in inpatients.

An alternative to reduce the costs associated with the prevention of VTE in the hospital is to update the protocol. Justified non-adherence generated savings due to the omission of one administration/day of UFH for patients at high risk, which is still effective for prophylaxis. Therefore, the Pharmacy and Therapeutic Commission could standardize the dose of heparin to twice a day instead of the three times described in the protocol.

## AUTHOR CONTRIBUTIONS

Mastroianni PC designed the study, aided with data collection, and wrote the manuscript. Oliveira ARA aided with data collection, data tabulation and discussion, and manuscript writing. Varallo FR aided with data collection, data tabulation and discussion, and manuscript writing. de Nadai TR and Forgerini M aided with data tabulation and discussion, and manuscript writing.

## Figures and Tables

**Table 1:** Criteria for indication of the therapeutic prophylaxis for VTE according to the clinical conditions of the patient and the care protocol.

Clinical condition	Indication of therapeutic prophylaxis according to protocol
Creatinine clearance <30 mL/minute	Preferably use UFH^a^ 5000 IU subcutaneously 12/12 h. If you use LMWH, use a 20 mg subcutaneous dose once daily
Morbid obesity (BMI>40)	Use UFH 7,500 IU 8/8 h
Full anticoagulation, heparin hypersensitivity, heparin- induced thrombocytopenia and active bleeding	Absolute contraindication for the use of prophylactic therapy
Recent intracranial or ocular surgery, CSF collection in the last 24 h, uncontrolled arterial hypertension (>180x110 mmHg)	Relative contraindication for the use of prophylactic therapy
Stroke	Preference for the use of LMWH

a UFH: unfractionated heparin; LMWH: low-molecular-weight heparin; BMI: body mass index; CSF: cerebrospinal fluid.

**Table 2:** Description, frequency and cost of adherence to the criteria of the prophylaxis therapy protocol for venous thromboembolism (VTE).

Description of protocol criteria	Patient (N)^a^	Prescription (N) (N)^b^	Cost of adherence ($)^c^
UFH^d^ 8/8 h for patient at high risk	49	343	1014.93
UFH 12/12 h for patient with clearance <30 mL/minutes	16	183	361.02
Nonreceipt of prophylaxis for patient with contraindication	13	25	0
UFH 12/12 h for average patient risk	01	13	25.65
LMWH 40 mg for high-risk patients with justification	03	06	20.58
UFH for low-risk patients with justification	0	0	0
UFH 7,500 IU 8/8 h for morbidly obese patients	0	0	0
LMWH 40 mg with justification (low-risk patients)	0	0	0
LMWH 40 mg with justification (medium-risk patients)	0	0	0
Total	50	457	1422.18

a Total number of patients.

b The total number of prescriptions is less than the sum of all items because the same patient and/or same prescription has been evaluated for more than one criterion.

c Value of the dollar: 1 dollar = 3.92 Brazilian Reais.

d UFH: unfractionated heparin; LMWH: low-molecular-weight heparin.

**Table 3:** Description, frequency, cost of non-adherence (with and without justification) and of referential adherence, and economic analysis, according to the criteria established in the prophylaxis protocol for venous thromboembolism (VTE).

	Patient (N)^a^	Prescription (N)	Cost of non-adherence ($)^b^	Cost of referential adherence ($)	Economic analysis ($)
**Description of justified non-adherence**					
UFH^c^ 12/12 h for patients at high risk^d^	54	520	1027.37	1541.06	513.69
**Description of non-justified non-adherence**					
UFH for patients at risk without justification	19	167	390.20	0	-390.20
LMWH 40 mg for high-risk patients without justification	13	31	109.95	91.87	-18.07
LMWH 40 mg and UFH 5000 IU for morbidly obese patients	04	33	101.71	195.60	93.89
LMWH 40 mg for low-risk patients without justification	02	07	24.05	0	-24.05
LMWH 40 mg for medium-risk patients without justification	02	02	6.87	3.95	-2.92
UFH 8/8 h for patients with clearance <30 mL / minutes	01	11	32.60	21.73	-10.87
Nonreceipt of prophylaxis without contraindications	26	59	0	154.11	154.11
UFH 8/8 h for medium-risk patients	0	0	N/A	N/A	N/A
Subtotal	63	231	665.38	467.26	-180.05
Total	112	751	1692.75	2008.31	333.64

a The total number of patients and prescriptions is less than the sum of all items because the same patient and/or the same prescription were evaluated for more than one criterion.

b Value of the dollar: 1 dollar = 3.92 Brazilian reais.

c UFH: unfractionated heparin; LMWH: low-molecular-weight heparin; N/A: not applicable.

d Geerts WH, Bergqvist D, Pineo GF, Heit JA, Samama CM, Lassen MR, Colwell CW. Prevention of venous thromboembolism: American College of Chest Physicians evidence-based clinical practice guidelines (8th edition). Chest 2008;133.
